# Antithrombotic Therapy After Transcatheter Aortic Valve Replacement: An Overview

**DOI:** 10.1016/j.shj.2022.100085

**Published:** 2022-09-15

**Authors:** Mathew N. Hindi, Mariama Akodad, Thomas Nestelberger, Janarthanan Sathananthan

**Affiliations:** a Centre for Cardiovascular and Heart Valve Innovation, St. Paul’s Hospital, University of British Columbia, Vancouver, Canada; b Cardiovascular Research Institute Basel (CRIB) and Department of Cardiology, University Hospital Basel, University of Basel, Basel, Switzerland

**Keywords:** Antiplatelet therapy, Antithrombotic therapy, Oral anticoagulation, Transcatheter aortic valve replacement

## Abstract

Transcatheter aortic valve replacement (TAVR) is an established procedure for the treatment of patients with severe aortic stenosis. The optimal antithrombotic regimen following TAVR, currently unknown and inconsistently applied, is impacted by thromboembolic risk, frailty, bleeding risk, and comorbidities. There is a quickly growing body of literature examining the complex issues underlying antithrombotic regimens post-TAVR. This review provides an overview of thromboembolic and bleeding events following TAVR, summarizes the evidence regarding optimal antiplatelet and anticoagulant use post-TAVR, and highlights current challenges and future directions. By understanding appropriate indications and outcomes associated with different antithrombotic regimens post-TAVR, morbidity and mortality can be minimized in a generally frail and elderly patient population.

## Introduction

Transcatheter aortic valve replacement (TAVR) is an established procedure for the treatment of patients with severe aortic stenosis (AS).^1^^,^^2^ However, TAVR may be associated with thromboembolic and bleeding complications, impacting patient prognosis.^3^, ^4^, ^5^, ^6^ Antithrombotic treatments are often prescribed to mitigate the risk of stroke/transient ischemic attacks (TIAs), valvular thrombosis, and myocardial infarction (MI) following TAVR. However, the risk of bleeding associated with these therapies has to be balanced in this highly comorbid population.^2^^,^^4^^,^^7^ Additionally, a large percentage of patients undergoing TAVR have comorbid diseases including atrial fibrillation (AF), coronary artery disease (CAD), or peripheral artery disease, complicating the development of a unique postprocedural antithrombotic strategy.^1^^,^^2^^,^^8^, ^9^, ^10^ Antithrombotic regimens can include antiplatelet therapy, anticoagulant therapy, or a combination of both. Historically, recommendations on antithrombotic treatment following TAVR have been developed but are largely built on expert consensus and opinion rather than rigorous, extensive evidence.^11^^,^^12^

This review aims to provide an overview of both thromboembolic and bleeding risks following TAVR, current evidence on antithrombotic strategies after TAVR, and current guidelines and future directions.

## Thromboembolic and Bleeding Events Following TAVR

Thromboembolic and bleeding events can occur following TAVR and are associated with poor prognosis. Patients typically at high risk for bleeding complications are often also at high risk for ischemic events. These events may be due to procedural factors and patient-specific factors.^1^ Thromboembolic complications post-TAVR can lead to significant morbidity and mortality and are important targets for antithrombotic regimens.^1^^,^^2^ These include cerebrovascular events (CVEs), MI, and clinical and subclinical valve thrombosis (SCVT) with bioprosthetic valvular deterioration (BVD).^1^ Early thromboembolic complications are usually related to the TAVR procedure.^1^^,^^3^^,^^13^ Certain factors, such as embolization of native valvular components, exposure to thrombogenic molecules in stenotic valves, flow stagnation, and factors associated with valve positioning, can create a prothrombotic environment leading to periprocedural events.^1^^,^^7^^,^^14^, ^15^, ^16^

### Cerebrovascular Complications

Acute clinical stroke and TIA are both associated with a high degree of morbidity and mortality. Based on the results of 2 large patient cohorts who underwent TAVR, CVE risk may be elevated up to 2 years post-TAVR but occurs most commonly within the first 30 days. Factors that may predispose patients to postprocedural CVEs include balloon postdilation, valve embolization, new-onset and chronic AF, peripheral vascular disease, and prior cerebrovascular disease. Major stroke is associated with significant mortality at 30 days and onward.^17^^,^^18^ In the recent low-risk trials, 1-year and 2-year stroke rates were low, reaching 1.2% and 2.4%, respectively, in the PARTNER 3 trial^19^ and 4.1% at 1 year in the Evolut low-risk trial.^20^ In the PARTNER 2 trial including intermediate surgical risk patients, all-stroke rate was 5.5% at 30 days, 8% at 1 year, and 9.5% at 2 years.^21^ Acute stroke or TIA (<1 day post-TAVR) is often attributed to procedural and technical factors, while the key factor contributing to subacute (1-30 days) and late (>30 days) events is comorbid AF.^1^^,^^17^ Patients with both new-onset atrial fibrillation (NOAF) and preexisting disease are at significantly higher risk to develop CVEs and have higher morbidity and mortality rates, which underlies the recommendation for chronic anticoagulation in this population.^1^^,^^22^^,^^23^ The prevalence of preexisting AF in the TAVR population is estimated at approximately 15% to 50%.^22^^,^^23^ About 10% to 30% of patients develop NOAF postprocedurally, which often goes undiagnosed.^1^^,^^22^, ^23^, ^24^ An analysis from the Society of Thoracic Surgeons/American College of Cardiology (STS/ACC) TAVR registry showed that older, female patients with higher STS scores and nontransfemoral access had higher rates of NOAF.^25^ A study by Seeger et al.^26^ showed that patients with AF had increased rates of bleeding, stroke, acute kidney injury, and all-cause death compared to those in sinus rhythm.

### Myocardial Infarction

MI is a less common postprocedural occurrence but can have a devastating impact on short- and long-term mortality and morbidity outcomes. MI prevalence after TAVR is only about 1% to 2% and is thought to be generally due to periprocedural myocardial injury caused by low output during the valve implantation.^1^^,^^27^ Other mechanisms which might contribute to acute coronary occlusion include low coronary ostia heights, small anulus, valve-in-valve (VIV) procedures with a low virtual valve-to-coronary distance, and coronary embolism. Nonetheless, CAD is a very common comorbidity among TAVR patients, estimated at 40% to 75%,^3^^,^^27^^,^^28^ and can affect outcomes, thus necessitating its inclusion in antithrombotic decision-making.^29^

### Bioprosthetic Valve Thrombosis

Another important thromboembolic complication post-TAVR is the occurrence of bioprosthetic valve thrombosis. Valve thrombosis can involve subclinical leaflet thrombosis and clinical leaflet thrombosis. Subclinical leaflet thrombosis is characterized by reduced leaflet motion and hypoattenuated leaflet thickening (HALT) without affecting transvalvular pressure gradients—it is often detected on high-resolution computed tomography (CT) incidentally and has uncertain clinical significance. On the other hand, clinical thrombosis involves elevated transvalvular pressure gradients with clinical events, such as the occurrence of stroke.^1^^,^^30^^,^^31^ The PARTNER 3 trial found that valve thrombosis was significantly higher in patients who underwent TAVR than those who underwent surgical replacement (2.6% vs. 0.7%, *p* = 0.02).^19^ The rate of clinical valve thrombosis and BVD has been found to be approximately 1% to 3% after TAVR, while SCVT is found in about 10% to 15% of patients at 30 days,^30^^,^^32^, ^33^, ^34^ and this increases to up to 30% at 1 year post-TAVR.^35^ Recent studies have suggested that subclinical leaflet thrombosis could be associated with poor outcomes including stroke, silent ischemic infarcts, and mortality.^36^, ^37^, ^38^ Risk factors for clinical leaflet thrombosis are VIV TAVR procedures, a balloon-expandable prosthesis, and the absence of anticoagulation, which has been shown to decrease and even reverse the risk of subclinical and clinical leaflet thrombosis by lowering transvalvular pressure gradients.^1^^,^^30^^,^^36^ VIV TAVR has emerged as a viable and less invasive option for patients with degenerated surgical valves and shows promise in improving short-term morbidity and mortality. However, studies have shown that VIV TAVR is associated with transprosthetic gradient progression over time and hemodynamic valve deterioration and thrombosis.^30^^,^^39^ The mechanism for this may be related to increased mechanical stress on the transcatheter heart valve (THV) leaflets and altered flow patterns.^30^^,^^39^ Unfortunately, most studies have not evaluated the optimal antithrombotic therapy for patients post-VIV TAVR, leaving the approach to the operator. While further studies are required, a more aggressive antithrombotic approach has been suggested in patients with low bleeding risk to combat the risk of clinical and SCVT; however, clinical and anatomical factors play a role in the decision-making process.^40^

### Bleeding Events Following TAVR

Bleeding risks, especially major and life-threatening bleeding, can complicate the development of antithrombotic regimens following TAVR. In the low-risk Evolut trial, approximately 2.4% and 3.2% of patients suffered life-threatening or disabling bleeding at 30 days and 12 months post-TAVR, respectively. These rates were also significantly lower than that for surgical patients.^20^ On the other hand, an analysis of the PARTNER 2 trial showed an incidence of life-threatening or disabling bleeding at approximately 10.4% at 30 days, 15.2% at 1 year, and 17.3% at 2 years post-TAVR in intermediate-risk patients.^21^ A meta-analysis showed that postprocedural bleeding increased the 30-day mortality by 323%, while major and life-threatening bleeding increased the mortality by 410%.^41^ Both early and late bleeding negatively impact cardiovascular (CV) and all-cause mortality.^1^^,^^40^^,^^20^ Early bleeding events (<30 days postprocedure) make up the majority of bleeding after TAVR and are usually related to procedural and technical factors associated with access site complications related to large sheath sizes but also to secondary access.^1^^,^^3^^,^^42^^,^^43^ On the other hand, late bleeding events (>30 days postprocedurally) are usually associated with nonaccess site complications, most frequently being gastrointestinal (GI) in origin.^42^^,^^44^ Late bleeding events have also been associated with a significant increase in mortality of about 3- to 4-fold.^42^^,^^44^ GI complications are most frequently associated with major late bleeding complications.^44^^,^^45^ AF, atrial flutter, diabetes mellitus, frailty (independent of age), anemia, hypertension, and renal impairment are well-known predictors of bleeding post-TAVR.^44^^,^^46^^,^^47^ According to the STS/ACC TAVR registry, vascular and bleeding complications were independently associated with an increased 30-day and 1-year mortality and rehospitalization.^48^

## Antithrombotic Regimen After TAVR

### Current Antithrombotic Guidelines

**Figure 1:**
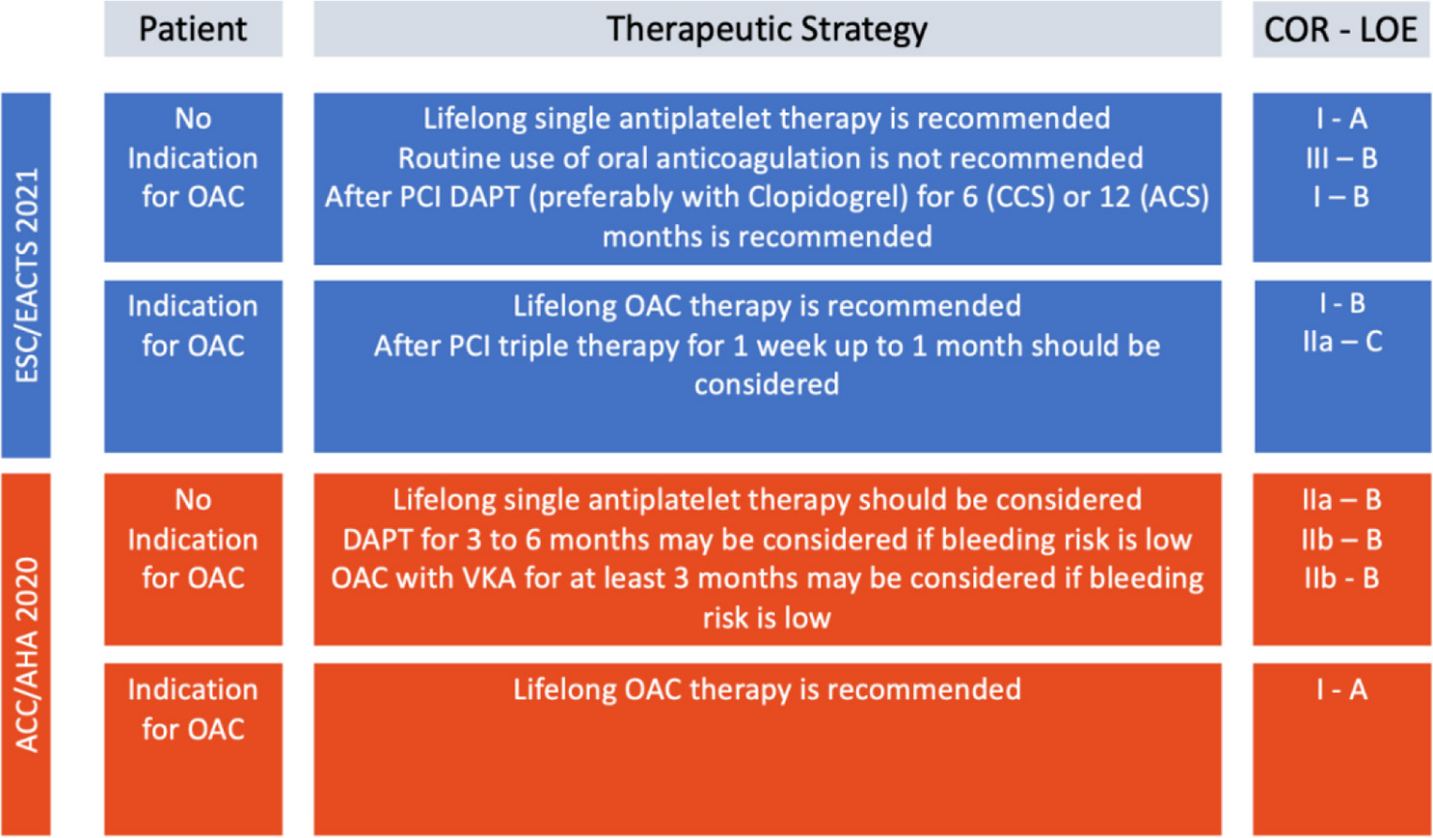
**Summary of current antithrombotic therapy guidelines after transcatheter aortic valve replacement.** Abbreviations: ACC/AHA, American College of Cardiology/American Heart Association; ACS, acute coronary syndrome; CCS, chronic coronary syndrome; COR, class of recommendation; DAPT, dual antiplatelet therapy; ESC/EACTS, European Society of Cardiology/European Association for Cardiothoracic Surgery; LOE, level of evidence; OAC, oral anticoagulant; PCI, percutaneous coronary intervention; VKA, vitamin K antagonist.

Antithrombotic strategies post-TAVR can range from a single antiplatelet regimen to dual antiplatelet therapies, to the use of oral anticoagulants (OACs) with or without concomitant antiplatelet therapy. Many of these guidelines are based largely on expert consensus and small, nonrandomized studies, which may explain the heterogenous antithrombotic approaches used in clinical practice.^1^ Furthermore, studies have found significant variability among medical centers.^3^^,^^12^^,^^49^, ^50^, ^51^, ^52^ However, this is a rapidly evolving field with many ongoing trials aiming to elucidate optimal antithrombotic regimens after TAVR. Current guidelines on post-TAVR antithrombotic strategies are summarized in Figure 1.

Patients after bioprosthetic surgical aortic valve replacement, on the other hand, typically require different antithrombotic regimens than TAVR, most often anticoagulation for 3 months’ duration according to the ACC/American Heart Association.^53^ In patients with an indication for anticoagulation, direct oral anticoagulant (DOAC) or vitamin K antagonist (VKA) is typically started for 3 to 6 months (depending on the indication for anticoagulation and other factors such as the risk of bleeding). If OAC therapy is discontinued, then acetylsalicylic acid is typically started at 75 to 100 mg daily subsequently. The same regimen may be used for patients without specific indication for anticoagulation but who are assessed to be of low bleeding risk; however, VKA is preferred over DOAC in these patients due to more data supporting this regimen to date. In patients with an elevated bleeding risk without indication for OAC, acetylsalicylic acid may be started at 75 to 100 mg daily (depending on the assessment of risk-to-benefit ratio).^53^

### Antiplatelet Therapies

Antiplatelet therapies are currently the basis of thromboembolic prophylaxis in patients without concomitant indication for anticoagulant therapy. Over the last decade, patients undergoing TAVR without indication for anticoagulation and no prior antiplatelet therapy were started on a variation of dual antiplatelet therapy (DAPT) with aspirin and clopidogrel (a thienopyridine∗ P2Y12 inhibitor† antiplatelet) for 3 to 6 months’ duration, followed by lifelong aspirin alone (single antiplatelet therapy or SAPT).^54^, ^55^, ^56^, ^57^ Many guidelines do not give specific or uniform information regarding patients with an indication for chronic anticoagulation.^54^, ^55^, ^56^, ^57^

Evidence regarding antiplatelet therapy following TAVR is based mainly on high platelet reactivity (HPR) found in patients with AS and after bioprosthetic valve implantation, as well as the interaction between the THV frame and platelets prior to endothelization. This interaction may be compared to that between stent and platelets in patients undergoing percutaneous coronary intervention.^5^^,^^58^^,^^59^ Moreover, around 70% of patients undergoing TAVR have concomitant CAD, thus guiding the use of antiplatelet therapy in these patients.^1^^,^^11^ DAPT for a few months followed by SAPT has been considered as the optimal strategy following TAVR. However, concerns about a potential higher bleeding risk of DAPT compared to SAPT has been raised leading to randomized trials comparing these strategies.

A recent randomized trial investigated the safety of 3 months SAPT with aspirin (n = 331) vs. DAPT with aspirin + clopidogrel (n = 334) in patients undergoing TAVR without indications for long-term OAC therapy.^55^ Bleeding events, mainly nonprocedure related, occurred in 50 patients on SAPT vs. 89 patients in the DAPT group (15.1% vs. 26.6%, *p* = 0.001). A composite of CV death, nonprocedural bleeding, stroke, or MI occurred in 76 and 104 patients, respectively (23.0% vs. 31.1%, *p* < 0.001). This study illustrates that in patients undergoing TAVR without an indication for OAC therapy, SAPT may provide a safer option over DAPT due to a reduction in bleeding complications and its noninferiority in preventing thromboembolic events.^60^ DAPT should mostly be reserved for patients with indications (such as recent stenting). This study was consistent with the Aspirin Versus Aspirin + Clopidogrel Following Transcatheter Aortic Valve Implantation (ARTE) randomized trial including 222 patients without indication for OAC therapy who underwent TAVR with a balloon-expandable valve.^61^ In this study, a composite end point of mortality, MI, stroke, and TIA tended to occur more frequently in the DAPT group vs. the SAPT group (15.3% vs. 7.2%, *p* = 0.065) at 3 months post-TAVR. Major and life-threatening bleeding was also significantly more frequent in the DAPT group compared to the SAPT group (10.8% vs. 3.6%, *p* = 0.038). A recent meta-analysis, comprising 4 randomized controlled trials (RCTs) and 1086 participants over a 9.1-month follow-up found similar results; it was found that DAPT increased the risk of major bleeding compared to SAPT, but they did not differ in the risk of stroke, MI, death, or cardiac death.^62^ Other recent studies and updated meta-analyses largely agree with the findings that post-TAVR DAPT therapy increases the risk of bleeding events without reducing the rates of thromboembolism and mortality compared to SAPT.^63^, ^64^, ^65^, ^66^ Even though these results are in agreement, larger studies with a longer mean follow-up time are required to adequately assess these end points more effectively.

Multiple studies analyzing data from the STS/ACC TAVR registry with over 16,000 patients has shown that DAPT therapy had a similar adjusted mortality, stroke, and MI risk at 1 year after TAVR compared with SAPT (aspirin or a P2Y12 inhibitor). However, the risk of major bleeding was significantly higher in patients treated with DAPT compared to SAPT.^67^ A systematic review and network meta-analysis by Zhu et al.^68^ evaluated the safety and efficacy of various antithrombotic strategies across 8 studies involving a total of 2173 patients. It was found that DAPT was associated with a higher 30-day bleeding risk but no change in all-cause mortality or stroke compared to SAPT therapy. Regarding the risk of leaflet thrombosis and HALT, DAPT has not shown any benefit compared to SAPT. In a study of 439 patients discharged on SAPT or DAPT therapy, outcomes including net adverse clinical events, all-cause mortality, CVEs, and valve thrombosis did not differ between the groups.^69^ Another systematic review of 1086 patients across 4 RCTs found that in patients without indication for anticoagulation, a composite of thromboembolism and bleeding occurred significantly less frequently in patients on aspirin alone compared with DAPT therapy. Aspirin alone did not increase the incidence of thromboembolism postprocedurally either.^70^ A meta-analysis by Al Halabi et al.^71^ including 8 studies and 2439 patients found that DAPT actually increased the risk of all-cause mortality as well as major and life-threatening bleeding and major vascular complications at 30 days without decreasing stroke, TIA, or MI rates. After 6 months, the increased bleeding risk remained.

A recent study was carried out on data from the Optimized transCathEter vAlvular iNtervention registry to determine the optimal SAPT strategy post-TAVR. It was found that while all-cause mortality did not differ between groups, monotherapy with clopidogrel was associated with a lower incidence of CV death compared with aspirin monotherapy, regardless of OAC use, over a 2-year period post-TAVR.^72^

The P2Y12 inhibitor ticagrelor has also been investigated. Assessment of Platelet REACtivity After Transcatheter Aortic Valve Replacement trial was a randomized clinical trial investigating the efficacy of clopidogrel and ticagrelor in suppressing HPR in 68 patients before TAVR. Ticagrelor (+aspirin) significantly reduced platelet reactivity at 6 ​hours, 24 ​hours, 5 days, and 30 days after TAVR compared to clopidogrel (+aspirin) therapy.^5^ No differences in major bleeding or CV death between groups were observed at 4-month follow-up. An analysis from the Everyday Practice With Transcatheter Aortic Valve Implantation database showed that patients with a status of low platelet reactivity had an increased risk of Valve Academic Research Consortium-2 (VARC-2) bleeding events compared to patients with HPR at 30 days after TAVR (45.6% vs. 23.9%, *p* = 0.01).^73^ HPR was not associated with increased risk of stroke, death, or MI at 30 days. Even though this study had a small sample size, thus limiting its clinical utility, it suggests that further studies are required to understand the value of platelet function for bleeding risk and antithrombotic therapy guidance. Caution should be taken when using more potent antiplatelet therapies, such as ticagrelor, in patients undergoing TAVR due to this potential bleeding risk. An important indication for the use of antiplatelet regimens is percutaneous coronary intervention. The duration of antiplatelet therapy in this context depends on the occurrence of stable CAD vs. acute coronary syndrome.^74^

Further studies examining optimal antiplatelet regimens after TAVR are ongoing. The TICTAVI trial is a randomized, open-label clinical trial.^75^ It hypothesizes that ticagrelor alone (180 mg loading dose before TAVR followed by 90 mg twice daily for 30 days post-TAVR) will be noninferior to aspirin with clopidogrel therapy on safety outcomes within 30 days after TAVR. Results from this trial will help clarify the safety and efficacy of various antiplatelet regimens.

### Anticoagulant Therapies

Evidence regarding anticoagulation therapy following TAVR is based mainly on the interaction between coagulation factors, thrombin, and THV leaflets and frame; the increased risk of postprocedural AF, flow turbulence through the valve, and vessel wall disruption lead to hemostatic activation.^2^^,^^3^^,^^58^ At present, OACs are only recommended for patients with other indications for anticoagulation (such as AF).

An RCT comparing the efficacy of aspirin vs. aspirin + warfarin in 94 patients found that the rate of HALT was 16.3% vs. 4.7% (*p* = 0.07), respectively, without excess bleeding at 30-day follow-up in low-risk patients post-TAVR.^76^ This study suggests that VKA anticoagulation post-TAVR may be effective in preventing THV dysfunction^76^ in the short postprocedural term without considerable early safety risks, but this necessitates longer term follow-up. Anticoagulation therapy has also been observed to reduce or prevent subclinical and clinical valve thrombosis in several observational studies using different doses and types of OACs.^31^^,^^60^^,^^74^, ^75^, ^76^, ^77^ In a meta-analysis by Woldendorp et al.^77^ including 3456 patients with 11.5% having SCVT, OAC therapy was found to be superior to SAPT or DAPT in protecting against SCVT (*p* < 0.0001). However, this study presents observational data, did not analyze bleeding outcomes, and did not have a uniform CT follow-up. Thus, further studies are needed to understand the safety and temporal associations between antithrombotic therapies and SCVT. Another meta-analysis by Rheude et al.^31^ found that OAC therapy was associated with a lower prevalence of BVD (*p* = 0.007). A nonrandomized study by Jimenez et al.^78^ was carried out on 90 patients assessing hypoattenuation affecting motion and HALT on post-TAVR CT. The study showed that a lack of OAC therapy was an independent predictor of SCVT. Further analyses showed that hypoattenuation affecting motion actually regressed after initiation of OAC therapy.^60^ According to an analysis of the French Transcatheter Aortic Valve Implantation registry, OAC therapy after discharge was associated with reduced rates of valvular deterioration and dysfunction.^79^ However, multivariable regression analysis revealed that anticoagulation at discharge, but not antiplatelet therapy, was associated with an increase in all-cause mortality. Bleeding and stroke outcomes were not analyzed in this study. Another study found that patients not discharged on OAC therapy had a mean aortic valve gradient increase >10 mmHg from 30 days to 1 year post-TAVR compared to those discharged on OAC.^80^ OAC was also independently associated with minor bleeding post-TAVR. Importantly, these studies suggest that anticoagulation has beneficial effects on valvular thrombosis and deterioration, unlike the use of antiplatelet therapy. A recent meta-analysis showed that patients receiving aspirin or OAC therapy with clopidogrel had approximately double the incidence of major bleeding, without affecting rates of mortality, MI, or stroke, compared to these regimens without clopidogrel.^81^ While further randomized studies are needed to better understand the efficacy of OAC therapies on SCVT, the potential increase in all-cause mortality and bleeding rates prevents the routine use of OAC therapy post-TAVR in patients without indication due to a problematic risk-reward relationship.

Recently, DOACs have been of interest due to their potential safety profile compared to VKA therapies. In general, studies have shown that DOACs are equivalent to VKA therapies in preventing thromboembolic complications but may confer a benefit by reducing bleeding rates. A study by Seeger et al.^26^ compared the safety and efficacy of apixaban (anti-Xa DOAC) vs. warfarin (VKA) in patients with AF after TAVR. Apixaban decreased the rate of the early safety end point compared to VKA therapy (13.5% vs. 30.5%, *p* < 0.01) at 30 days and led to a significantly lower rate of life-threatening bleeding. There was also a trend toward lower stroke rate at 30 days with apixaban. A prospective, multicenter, observational cohort registry with data from 403 patients with AF post-TAVR showed that DOAC use was associated with a lower all-cause mortality compared with the use of VKA (10.3% vs. 23.3%, *p* = 0.005) in patients with severe AS who were successfully discharged after TAVR at 720 days of follow-up.^82^ Conversely, a meta-analysis by Ueyama et al.^83^ showed that in patients post-TAVR with concomitant OAC indication, all-cause mortality, major and life-threatening bleeding, and stroke rates did not differ between DOAC and VKA groups, which was also shown in another meta-analysis by Butt et al.^84^ Analyses of data from the French Transcatheter Aortic Valve Implantation and a prospective multicenter study of the French national transcatheter aortic-valve implantation registry registries (including 8962 patients treated with OAC therapy) found that major bleeding and mortality were less prevalent in patients on DOAC therapies at 3 years compared to those on VKAs. Ischemic stroke and acute coronary syndrome rates did not differ significantly between the groups.^85^ Analysis of data from the Vienna Registry (VIenna CardioThOracic Aortic Valve RegistrY trial) showed a trend toward improved 3-year survival in patients on non-VKA OACs compared to those on a VKA regimen.^86^ Additionally, a recent study involving over 21,000 patients enrolled in the STS/ACC Transcatheter Valve Therapy Registry registry showed that in patients post-TAVR with AF, DOAC use has a comparable stroke risk with lower incidence of bleeding, intracranial hemorrhage, and death at 1 year compared with VKA therapy.^87^ While much of the literature suggests that DOAC therapy is a potentially safe alternative to VKA therapy, there are still only a small number of nonrandomized studies examining this. Thus, further RCTs will need to be done to better understand the safety and efficacy of different DOAC regimens and their appropriate dosage schedules. There are currently several ongoing trials investigating the safety and efficacy of DOAC therapies.

Recent data from the Edoxaban versus Standard of Care and Their Effects on Clinical Outcomes in Patients Having Undergone Transcatheter Aortic Valve Implantation–Atrial Fibrillation multicenter randomized trial has further explored this area. The study compared the efficacy of edoxaban (a DOAC inhibiting factor Xa) and VKA therapy in patients with AF after successful TAVR. While the rates of the composite primary outcome, stroke, and all-cause death did not differ significantly between groups, the incidence of major bleeds (mainly GI in nature) were higher in the edoxaban group compared to the VKA group.^88^ Recent findings from the Anti-Thrombotic Strategy After Trans-Aortic Valve Implantation for Aortic Stenosis multicenter randomized trial found that the composite of death, MI, and stroke, as well as the bleeding outcomes and valve thrombosis rates, did not differ between patients receiving apixaban and those receiving a VKA (for patients requiring OAC therapy).^89^

The Global Study Comparing a rivAroxaban-based Antithrombotic Strategy to an antipLatelet-based Strategy After Transcatheter aortIc vaLve rEplacement to Optimize Clinical Outcomes investigated the efficacy and safety of a 10 mg daily dose of rivaroxaban (with 75-100 mg aspirin for the first 3 months) vs. 75 to 100 mg daily aspirin (with 75 mg clopidogrel for the first 3 months) in patients without indication for OAC therapy.^90^^,^^91^ The trial was terminated early due to the finding that the rivaroxaban group had a higher risk of all-cause death, thromboembolism, and bleeding. The 10 mg dose in this trial was chosen to prevent valve thrombosis but avoid bleeding complications. However, this is less than what is usually prescribed for stroke prophylaxis in patients with AF^90^; thus, it is possible that this dose of rivaroxaban was not sufficient to have adequate therapeutic effect. As shown in the Comparison of a Rivaroxaban-based Strategy With an Antiplatelet-based Strategy Following Successful TAVR for the Prevention of Leaflet Thickening and Reduced Leaflet Motion as Evaluated by Four-dimensional, Volume-rendered Computed Tomography substudy, the 10 mg rivaroxaban-based therapy is more effective than the antiplatelet-based strategy in preventing SCVT and leaflet motion abnormalities.^92^ Based on these results, an anticoagulation strategy should not be the default therapy for patients who do not have any other indication for anticoagulation. It is possible that a lower dose of DOAC than required for stroke prevention may be sufficient for the prevention of valvular dysfunction, but further studies are needed to confirm this.

Further studies to elucidate the safety and efficacy of various antithrombotic regimens are currently underway. The Anticoagulant Versus Dual Antiplatelet Therapy for Preventing Leaflet Thrombosis and Cerebral Embolization After Transcatheter Aortic Valve Replacement trial is an active multicenter, randomized, open-label controlled clinical trial comparing the efficacy of a DOAC (edoxaban 60 mg daily for at least 6 months) vs. DAPT (clopidogrel 75 mg plus aspirin 75-100 mg for at least 6 months) in preventing leaflet thrombosis and cerebral embolization.^93^ The Rotterdam EDOXaban Leaflet Evaluation in Patients After Transcatheter Aortic Valve Implantation trial is a single-center, open-label phase III clinical trial with single-group assignment that is investigating whether treatment with 3 months of edoxaban after TAVR is safe and effective in reducing leaflet thickening.^94^

### Combination of Antiplatelet and Anticoagulant Therapies

Following TAVR, antiplatelet therapy in combination with anticoagulation may be required for patients with indication for chronic OAC therapy. Indeed, AF is a common comorbidity in patients undergoing TAVR and is classically associated with thromboembolic complications and mortality.^26^

A study by Geis et al.^95^ investigated the use of antiplatelets with OAC therapy in 167 patients with AF undergoing TAVR. Patients received VKA monotherapy, VKA + SAPT, or VKA + DAPT regimen, and it was found that patients on VKA monotherapy had significantly lower rates of major or life-threatening bleeding than those also on antiplatelets without increased thromboembolic risk. Additionally, a recent meta-analysis of 4 studies including 2032 patients post-TAVR with concomitant AF has shown that major and life-threatening bleeding rates are lower in patients on an OAC regimen compared to OAC + antiplatelet regimens, while the risk of stroke and mortality do not differ between the groups.^96^ Similar results were demonstrated in a meta-analysis by Navarese et al.,^64^ showing that OAC therapy alone reduced bleeding rates compared to OAC + SAPT regimens, without affecting stroke rates, in patients requiring long-term anticoagulation. On the contrary, another study of the PARTNER 2 trial by Kosmidou et al.^97^ showed that in patients with a history of AF, OAC therapy reduced the risk of death and stroke at 2 years post-TAVR only if combined with antiplatelet regimens.

A study by Zhu et al.^68^ showed that DAPT + OAC therapy (triple therapy) has been associated with a higher 30-day bleeding risk compared to DAPT, SAPT, OAC, and SAPT + OAC therapies. Triple therapy also ranked as the worst regimen for all outcomes (bleeding, stroke, and mortality measures). Another meta-analysis by Kuno et al.^98^ included 3 RCTs and 10 nonrandomized studies, with a total of 20,548 patients. They showed that SAPT was associated with lower bleeding rates compared to DAPT, SAPT + OAC, and DAPT + OAC, with no significant differences in stroke risks. OAC + DAPT triple therapy had higher rates of mortality compared with the other regimens.

Thus, the majority of literature suggests that in patients with AF and without indication for antiplatelet regimens, OAC therapy alone may be a safe and effective regimen. In addition, in patients with indication for OAC therapy, triple therapy with DAPT + OAC is associated with a prohibitive bleeding risk.^64^^,^^95^ The benefit of combining antiplatelets with OAC regimens post-TAVR is not proven and may harbor safety risks.^99^ Whether this is an acceptable therapy post-TAVR, especially in those with concomitant CAD, is not clear and still under investigation.^100^

Further studies examining the safety and efficacy of combining antiplatelet regimens with anticoagulant therapies are currently underway. The Anticoagulation Alone Versus Anticoagulation and Aspirin Following Transcatheter Aortic Valve Interventions trial is a multicenter, randomized, open, controlled trial that hypothesizes that a single anticoagulant alone is superior to the combination of an anticoagulant with an antiplatelet in terms of clinical benefit at 12 months post-TAVR based on bleeding complications (Bleeding Academic Research Consortium-2 [BARC-2] criteria) and other complications (VARC-2 criteria) including all-cause death, MI, all-cause stroke, valvular thrombosis, and hemorrhage.^101^ The LRT 2.0 trial is an active randomized, open-label clinical trial where patients are randomized to receive warfarin (a VKA anticoagulant) with aspirin for 30 to 45 days post-TAVR or aspirin alone post-TAVR. The study attempts to investigate the optimal antithrombotic therapy in low-risk patients.^102^

Data examining the outcomes for patients not on any antithrombotic therapy both in the immediate and long-term setting after TAVR are very scarce. In a few studies, a subgroup of patients with severely high bleeding risk or other comorbidities that contraindicate antithrombotic therapy have been studied. A subanalysis of the PARTNER 2 trial^80^ revealed that about 4% of the patients in the TAVR arm (3889 patients total) did not receive any antithrombotic therapy; however, no analyses were reported for this group due to a small sample size. Another study analyzing data from the VIenna CardioThOracic Aortic Valve RegistrY trial found that patients receiving no treatment (20 patients) had similar overall CV and cerebrovascular mortality between 1 and 5 ​years of follow-up when compared to groups receiving SAPT (172 patients), DAPT (34 patients), and OAC (163 patients) therapy.^86^ Due to the small sample size, no further analysis was possible for this patient cohort.

## Future Directions

**Figure 2:**
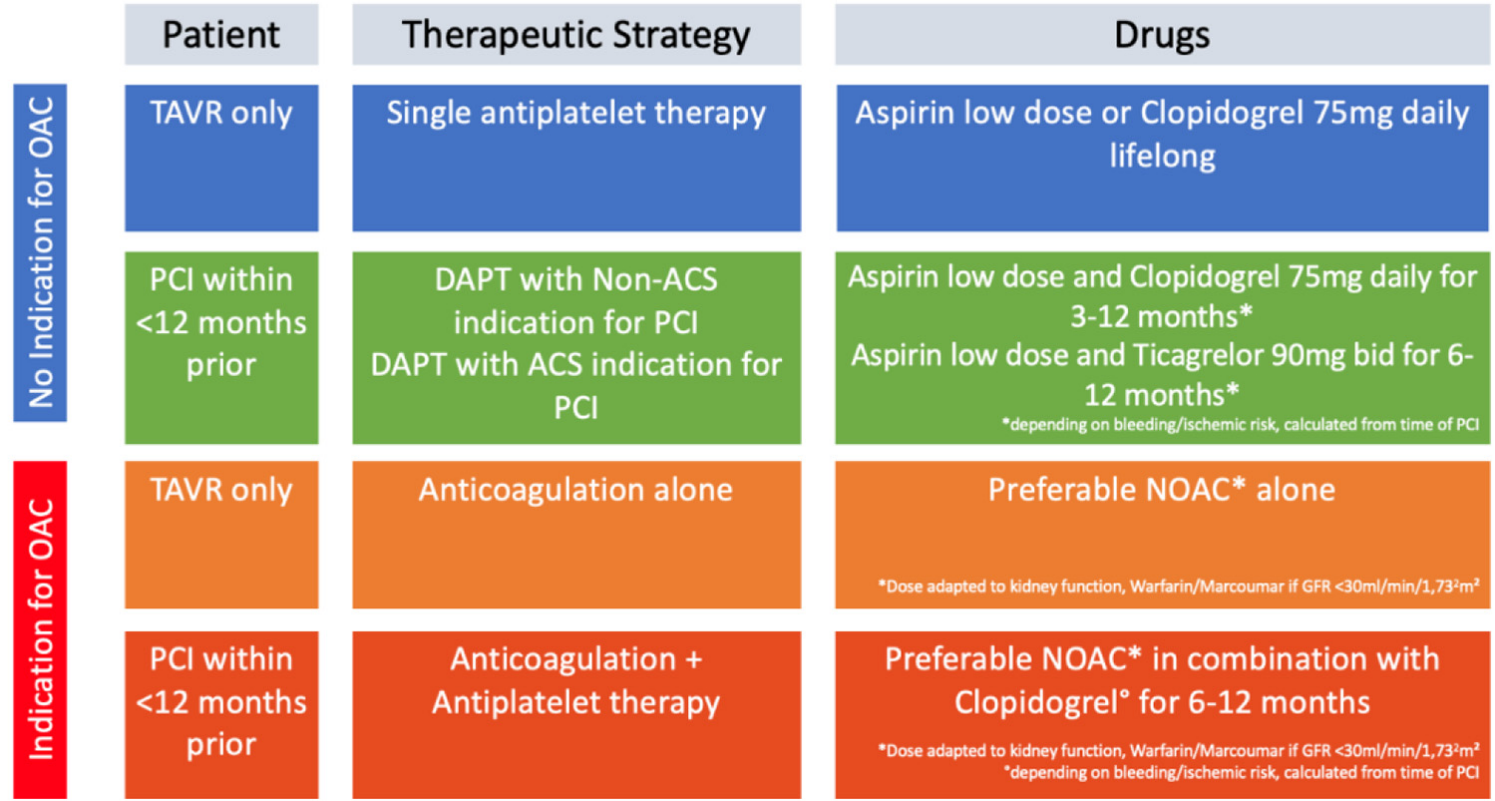
**Suggested antithrombotic regimen after Transcatheter aortic valve replacement (TAVR).** Abbreviations: ACS, acute coronary syndrome; DAPT, dual antiplatelet therapy; NOAC, novel oral anticoagulant; OAC, oral anticoagulant; PCI, percutaneous coronary intervention.

The development and improvement of TAVR has resulted in major benefits for patients with regard to mortality, morbidity, and quality of life. The antithrombotic regimen after TAVR, however, has mostly remained an unexperienced field. The current practice guidelines are largely based on expert opinions and followed variably around the world. This also resulted in diverging guidelines, as outlined in Figure 1. Further studies are required to better understand the optimal antithrombotic therapies following TAVR in order to optimize patient outcomes and inform clinical guidelines. Key ongoing clinical studies are summarized in Table 1. Antithrombotic therapy following TAVR requires a tailored approach. Based on the current data, we have tried to provide a simplistic, safe, and efficient recommendation on antithrombotic therapy after TAVR in Figure 2.

**Table 1:** Summary of current ongoing clinical studies

Trial	N#	Test arm	Control arm	Duration	Primary completion date	Primary endpoint
TICTAVI (NCT02817789)	308	Ticagrelor alone	ASA + clopidogrel	30 d post-TAVR	May 2018	VARC-2 composite endpoint (all-cause death, all stroke, life-threatening or disabling bleeding, acute kidney injury , CAD, major vascular complication, and valvular dysfunction requiring repeat-TAVR)
ADAPT-TAVR (NCT03284827)	245	Edoxaban	ASA + clopidogrel	6 mo post-TAVR	October 2021	Incidence of leaflet thrombosis (as assessed by 4DCT)
ENVISAGE-TAVI AF (NCT02943785)	1400	Edoxaban	VKA	36 mo post-TAVR	June 2021	NACE (all-cause death, MI, ischemic stroke, systemic embolic events, valve thrombosis, and major bleeding)
AVATAR (NCT02735902)	170	VKA or DOAC (apixaban or edoxaban)	ASA + VKA/DOAC	12 mo post-TAVR	April 2021	Composite of death, stroke, MI, valve thrombosis, and hemorrhage (as defined by VARC 2)
REDOX TAVI (NCT04171726)	100	Edoxaban	N/A	3 mo post-TAVR	December 2021	Incidence of leaflet thickening (as assessed by 4DCT)
LRT 2.0 (NCT03557242)	124	ASA + warfarin	ASA alone	30 d post-TAVR	July 2023	All-cause death, all stroke, life-threatening and major bleeding, major vascular complications, hospitalization for valvular symptoms or worsening congestive heart failure, HALT, at least a moderate degree of RELM, and hemodynamic dysfunction
PARTNER 3 (NCT02675114)	1000	TAVR with SAPIEN 3 THV	SAVR with commercially available bioprosthetic valve	12 mo post-TAVR	November 2020	All-cause mortality, all stroke and rehospitalization

4DCT, cardiac 4D computed tomography; ASA, acetylsalicylic acid; CAD, coronary artery disease; DOAC, direct oral anticoagulant; DVT, deep vein thrombosis; HALT, hypoattenuated leaflet thickening; HITS, high intensity transient signals; NACE, net adverse clinical events; PE, pulmonary embolism; RELM, reduced leaflet motion; SAVR, surgical aortic valve replacement; TAVI, Transcatheter Aortic Valve Implantation; TAVR,Transcatheter aortic valve replacement; THV, transcatheter heart valve; VARC 2, Valve Academic Research Consortium-2; VKA, vitamin K antagonist.

## Conclusion

The development of an optimal antithrombotic regimen after TAVR is important and complex due to the highly frail and comorbid patient population and the potential postprocedural complications including both ischemic and bleeding events. Many of the current recommendations regarding antithrombotic regimen are not based on high quality evidence but are rather based on expert consensus and opinion. Currently, while most guidelines recommend the use of DAPT for patients without indication for chronic OAC therapy, much of the recent evidence suggests that SAPT provides a safer and noninferior approach. Additionally, many studies have shown that while OAC therapy is superior to antiplatelet regimens in preventing subclinical and clinical valve thrombosis, therapy should only be a routine recommendation for patients with indication for chronic anticoagulation due to a higher bleeding risk. Furthermore, while DOACs represent a potential safer and noninferior approach to VKA anticoagulants, results regarding safety and efficacy are limited and mixed. Further studies will hopefully help clarify optimal antithrombotic therapies after TAVR for a diverse patient population.

## Funding

This research did not receive any specific grant from funding agencies in the public, commercial, or not-for-profit sectors.

## Disclosure statement

We disclose that Dr Nestelberger has received research support from the Swiss National Science Foundation (P400PM_191037/1), the Prof Dr Max Cloëtta Foundation, the Margarete und Walter Lichtenstein-Stiftung (3MS1038), and the University Hospital Basel, as well as speaker honoraria/consulting honoraria from Siemens, Beckman Coulter, Bayer, Ortho Clinical Diagnostics, and Orion Pharma, outside the submitted work. J. Sathananthan is a consultant to Edwards Lifesciences and Medtronic. Dr Akodad has received research funding from Medtronic, Biotronik MUSE Explore, and Federation Française de Cardiologie. Dr Sathananthan is a consultant to Edward Lifesciences and Medtronic. The other authors had no conflicts to declare.

## Footnotes

∗ Thienopyridine antiplatelets include clopidogrel and prasugrel.

† P2Y12 inhibitors include clopidogrel, ticagrelor, prasugrel, and cangrelor.
